# Analysis of Differences in Volatile Components of Five Lauraceae Plants From Different Genera Based on HS-SPME-GC-MS

**DOI:** 10.1155/jamc/9045731

**Published:** 2025-08-08

**Authors:** Zhengjiu Wang, Hao Liu, Anping Liu, Peng Liu, Jiuyang Zhao, Shaoli Fan, Jinhui Wang, Bahetiyar Keremu, Lu Yang, Le Li

**Affiliations:** ^1^Key Laboratory of Xinjiang Phytomedicine Resource and Utilization, Shihezi University, School of Pharmacy, Ministry of Education, Shihezi, China; ^2^Key Laboratory of Forest Resources and Utilization in Xinjiang of National Forestry and Grassland Administration, Xinjiang Academy of Forestry, Urumqi, China; ^3^Xinjiang Association for Science and Technology, Urumqi, Xinjiang, China

**Keywords:** chemotaxonomy, HS-SPME-GC-MS, Lauraceae, response surface, volatile components

## Abstract

Lauraceae plants are diverse in species and rich in volatile components, which possess functions such as insect repellency, antioxidant activity, and antibacterial properties. However, currently, the methods for analyzing the volatile components of Lauraceae plants are relatively single. The essential oils are mainly extracted by steam distillation, but the pretreatment is relatively complex and cumbersome. Therefore, it is essential to find a simple and cost-effective method. By comparing different extraction methods, HS-SPME-GC-MS was selected as the optimal extraction condition. Regarding Head-space Solid-Phase Microextraction and Gas Chromatography-Mass Spectrometry (HS-SPME-GC-MS), single-factor condition optimization and response surface analysis were carried out for different fiber coatings, equilibrium time, extraction temperature, and extraction time. Eventually, 75-μm CAR/PDMS fiber head was chosen, with an equilibrium time of 15 min, and extraction was conducted at 70°C for 57 min as the optimal HS-SPME extraction conditions. Furthermore, a differential analysis of the volatile components of five Lauraceae plants from different genera was performed, and differential metabolites were screened out respectively. This can effectively separate *Cinnamomum* and *Litsea* from the other three genera, providing certain assistance for the chemotaxonomy of the volatile components of Lauraceae plants and the subsequent development of medicinal plant resources.

## 1. Introduction

The Lauraceae family refers to a group of plants within the class Dicotyledoneae and the subclass Magnoliidae [1]. Most of them are trees or shrubs, and a large number of species within this family possess oil-containing cells in their fruits and leaves, from which a substantial quantity of volatile components can be extracted. These components mainly consist of linalool, citral, camphor, cinnamaldehyde, eucalyptol, and so on. These volatile substances exhibit functions like repelling insects and mosquitoes [2], antioxidation and anti-inflammation [3, 4], as well as antibacterial and antiviral effects [5–7]. Lauraceae plants can be categorized into around 45 genera, with a distribution across tropical and subtropical regions. They show remarkable diversity in China. In the recently published “Inventory of Higher Plants in China”, a total of 513 species belonging to 25 genera, including subspecies groups, have been recorded. Lauraceae plants are abundant in various chemical components, including aliphatic and aromatic aldehydes, ketones, alkenes, monoterpenes, and sesquiterpenes, all of which possess certain medicinal value [8]. Lauraceae plants are rich in aliphatic and aromatic aldehydes, ketones, alkenes, monoterpenes, and sesquiterpenes chemical components, which have certain medicinal value [9]. At present, cinnamon is the Lauraceae plant with the most thoroughly studied medicinal properties. Research has revealed that cinnamon branches are rich in compounds such as cinnamic acid amides and cinnamaldehyde [10], which can be used to treat microbial infections [11]. Moreover, cinnamon essential oil has a significant antibacterial effect [12], and has been used in the development and utilization of antibacterial composite films, significantly inhibiting the growth of *Bacillus* cereus, *Staphylococcus aureus*, *Escherichia coli*, and *Salmonella typhimurium* [13]. In addition, cinnamon extracts can inhibit the proliferation of tumor cells by upregulating pro-apoptotic molecules, and research has proven that it has strong anticancer ability [14]. The volatile oil of *Lindera aggregata* has been found to alleviate cardiomyopathy caused by diabetes in mice by inhibiting the MAPK/ATF6 pathway [15]. The sesquiterpenoids of *Litsea cubeba* have a certain inhibitory effect on cancer cells [16], and *Laurus nobilis* also has certain effects in helping sleep and protecting the liver [17]. Evidently, this group of plants holds significant medicinal value.

China abounds in species resources of Lauraceae plants. Nevertheless, currently, our comprehension of Lauraceae plants is predominantly confined to the research on the chemical components and medicinal properties of certain medicinal plants. There is a dearth of systematic research on the chemical components of Lauraceae plants in general, and numerous nonmedicinal plants await development and utilization. Thus, it is imperative to detect the volatile components of nonmedicinal Lauraceae plants. Our anticipation is to discover species that can substitute for medicinal plants during the processes of component identification and chemotaxonomy. At present, the principal research methods still center around the GC-MS identification of volatile oils, including techniques like the steam distillation method and the Soxhlet extraction method [18]. Although these methods boast high accuracy, are widely acknowledged by the majority of researchers, and are of vital importance in content determination, their intricate sample-processing procedures are time-consuming and labor-intensive. This poses a rather significant challenge for the qualitative research on the chemical components of a large quantity of Lauraceae plants. Consequently, it is extremely essential to seek a relatively simple and convenient detection method.

Odor detection methods such as Head-space Solid-Phase Microextraction and Gas Chromatography-Mass Spectrometry (HS-SPME-GC-MS), Automatic Thermal Desorption-Gas Chromatography-Mass Spectrometry (ATD-GC-MS), and Electronic nose were initially widely applied in the research of the environmental science field [19], Due to their high sensitivity in detecting volatile components, they have also been applied in the food and pharmaceutical fields in recent years. For example, HS-SPME-GC-MS has been used in the monitoring of food production and processing, such as flaxseed baking [20] and the dynamic change of green tea aroma [21]. It has also been involved in the pharmaceutical field, such as the detection of tangerine peel aging [22] and the metabolomics research of Houttuynia cordata [23]. This demonstrates that HS-SPME-GC-MS has certain research potential in the development of medicinal plants mainly composed of volatile components and is suitable for the analysis of a large number of chemical components. In the realm of volatile component differential analysis, there exists a broad spectrum of opportunities for further exploration. For example, by examining the variations in terpenoid constituents between green and red Sichuan peppercorns and employing chemometric techniques, one can effectively differentiate among various cultivars and geographical origins of these spices [24]. Moreover, conducting comparative analyses of chemical constituents from distinct plant parts—such as the leaves, stems, and flowers of *Pogostemon cablin*—has revealed notable differentiation efficacy through differential analysis and metabolic pathway investigations [25]. These analytical approaches are equally applicable to HS-SPME-GC-MS methodologies, thereby furnishing novel research avenues for the taxonomic classification of Lauraceae species. Therefore, in this research project, different extraction methods of volatile components will be screened. Based on the detection abundance and the number of compounds by GC-MS, the practicality of HS-SPME-GC-MS in the qualitative analysis of volatile components of Lauraceae plants will be verified, and the conditions of SPME will be optimized. The aim is to screen out the most suitable HS-SPME-GC-MS extraction conditions for most Lauraceae plants, which can be used for the qualitative detection of volatile components of Lauraceae plants, laying a prerequisite for the subsequent chemotaxonomy of the collected Lauraceae plants and the development of medicinal plant resources.

## 2. Materials and Methods

### 2.1. Materials and Reagents

The cassia twig used in the experiment is packaged decoction pieces from a pharmacy, sourced from Rong County, Guangxi Zhuang Autonomous Region, and complies with the implementation standards of Part 1 of the Chinese Pharmacopoeia (2020 Edition). The extraction solvents used are HPLC-grade *n*-hexane (imported from Germany, SIMARK) and HPLC-grade acetone. Anhydrous sodium sulfate is provided by Tianjin Zhiyuan Chemical Co., Ltd. The instruments used in the experiment are as follows: 7890A/5975C Gas Chromatograph-Mass Spectrometer (GC-MS), produced by Agilent Technologies; BSA224S Electronic Analytical Balance, provided by Sartorius Scientific Instruments (Beijing) Co., Ltd; DC-200 High-speed Multifunctional Crusher, manufactured by Zhejiang Wuyi Dingcang Daily-use Metal Factory; KMD Regulable Temperature Electrothermal Mantle, produced by Shandong Juancheng Hualu Electrothermal Instrument Co., Ltd; KQ-500DE Ultrasonic Cleaner, manufactured by Kunshan Ultrasonic Instrument Co., Ltd; C-MAG HS4 Magnetic Stirrer, from IKA Group, Germany; and 57318-type 50/30-μm DVB/CAR/PDMS, 65-μm PDMS/DVB, 75-μm CAR/PDMS, and 100-μm PDMS SPME fiber heads, provided by Supelco, USA.

### 2.2. Comparison of Different Extraction Methods

#### 2.2.1. Ultrasound-Assisted Extraction

Accurately weigh 0.5 g of the prepared powdered herbal sample (sieved through a 60-mesh screen) and transfer it into a 10-mL centrifuge tube. Add 5 mL of two extraction solutions (*n*-hexane and acetone), mix thoroughly, and allow it to soak for 6 h. Perform ultrasonic extraction for 50 min with the aid of a water bath, followed by centrifugation at 10,000 r/min for 5 min. Collect the supernatant and set it aside for further use.

#### 2.2.2. The Volatile Oil Extraction Method

The extraction was performed according to Method A in the “Determination of Volatile Oils” outlined in the Chinese Pharmacopoeia (2020 edition, General Rule 2204, Part IV). Accurately weigh 10 g of the powdered sample and soak it in distilled water at a ratio of 10:1 (distilled water to sample) for 1 h. The volatile oils were extracted using a volatile oil extraction apparatus, with distillation carried out for 6 h until no further increase in oil volume was observed in the extractor. The oil was extracted three times with petroleum ether. The combined petroleum ether extracts were dehydrated using anhydrous Na_2_SO_4_, followed by filtration. The filtrate was subjected to solvent recovery using a rotary evaporator, yielding a yellow volatile oil with a characteristic aroma. The oil was diluted with *n*-hexane and set aside for further use.

#### 2.2.3. HS-SPME Extraction

Accurately weigh 0.5 g of the prepared powdered herbal sample and transfer it into a 15-mL extraction vial, and then seal the vial. Place the extraction vial onto the SPME apparatus. After aging the SPME fiber for 5 min, insert the fiber through the vial cap into the headspace above the sample, positioning the fiber approximately 1.0 cm above the surface of the sample. Set the stirring speed to 150 r/min and allow the system to equilibrate for 15 min. The extraction was conducted for 40 min at 60°C. After sampling, insert the SPME fiber into the GC-MS injection port, desorb the analytes at 250°Cfor 5 min, and proceed with GC-MS analysis.

### 2.3. GC-MS Conditions

#### 2.3.1. Gas Chromatography Method

The sample analysis was performed utilizing a 7890A-5975C GC-MS system from Agilent Technologies, USA. The chromatographic column employed was a TR-5MS (60 m × 0.25 mm, 0.25 μm). High-purity helium (purity ≥ 99.999%) served as the carrier gas. The temperature program was established as follows: the initial temperature was set at 50°C, followed by an increase at a rate of 10°C/min to 160°C, where it was maintained for 10 min; subsequently, the temperature was raised at a rate of 1°C/min to 161°C and held for 5 min, and finally, the temperature was ramped up at 20°C/min to 260°C and held for 2 min, resulting in a total run time of 2 min. With the exception of HS-SPME injections, *n*-hexane was utilized as the solvent for all other samples, with a solvent delay of 5 min.

#### 2.3.2. Mass Spectrometry Parameters

The mass spectrometer model was 5975C Network MSD (Agilent Technologies). The following parameters were set for the MS: ionization source: electron impact (EI) with an energy of 70 eV; MS interface temperature: 250°C; ion source temperature: 230°C; quadrupole temperature: 150°C; acquisition mode: full scan; and mass scan range: m/z 40–650.

### 2.4. Screening of the Different Extracted Fibers

Volatile organic compounds from *Cinnamomum* cassia were extracted using four types of SPME fibers: 50/30 μm DVB/CAR/PDMS, 65 μm PDMS/DVB, 75 μm CAR/PDMS, and 100 μm PDMS. A 0.5-g sample of *Cinnamomum* cassia was weighed and placed in a 15-mL extraction vial, with the temperature of the magnetic stirrer set to 80°C and the stirring speed set to 150 rpm. The SPME fibers were exposed to the headspace of the vial for 50 min to adsorb the volatile organic compounds, which were then analyzed by SPME. The fibers were subsequently desorbed at 250°C for 5 min, followed by GC-MS separation and identification.

### 2.5. Single-Factor Condition Optimization

#### 2.5.1. Extraction Temperature

Extraction was carried out at temperatures of 20°C, 40°C, 60°C, 80°C, and 100°C, while the remaining conditions were kept consistent with the method outlined in Section 2.4.

#### 2.5.2. Extraction Time

The extraction process was conducted over durations of 20, 30, 40, 50, and 60 min, with all other parameters held constant in accordance with the methodology outlined in Section 2.4.

#### 2.5.3. Equilibrium Time

The extraction was performed with equilibrium times set to 10, 15, 20, 25, and 30 min, while the other conditions remained consistent with the method described in Section 2.4.

### 2.6. Response Surface Methodology Experiment

Based on the results obtained from the single-factor experiments, the extraction temperature, extraction time, and equilibrium time were selected as the influencing factors according to the principles of Box–Behnken design in central composite experiments. The factors and their levels are shown in Table 1.

### 2.7. Analysis and Identification of VOCs

The NIST 11 library in the GC-MS software was used to automatically search and analyze the mass spectral data of the components. All results were searched, and the data were checked and supplemented by referencing relevant standard spectra. The composition of each volatile compound was determined qualitatively, and the relative percentage content of each component in the total volatile substances was calculated using the total ion chromatogram (TIC) peak area normalization method.

### 2.8. Differential Analysis of Lauraceae Plants in Five Different Genera

According to the above optimization method, the representative plants of five different genera were analyzed, and the differences of metabolites were analyzed by stoichiometry, and the different metabolites were classified and analyzed.

## 3. Results and Discussion

### 3.1. Comparison of Different Extraction Conditions

**Figure 1:**
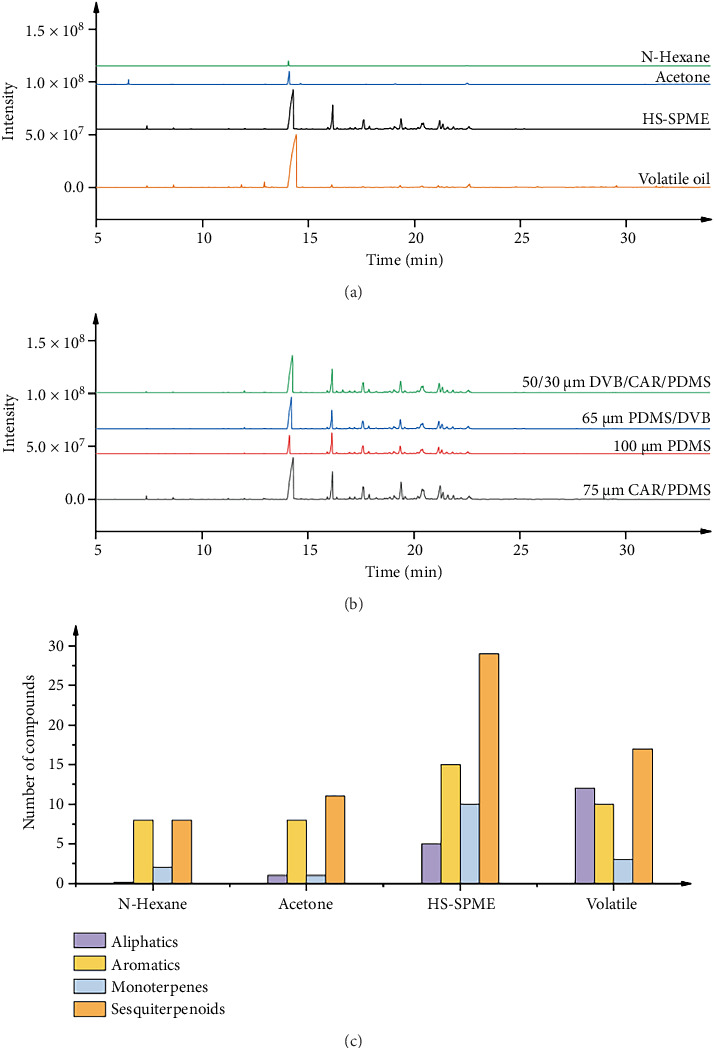
Condition optimization diagram of the screening method. (a) Comparison of TIC diagrams under different extraction conditions. (b) Comparison of TIC diagrams under different extraction fiber conditions. (c) Compound components under different extraction conditions.

Figure 1(a) shows a comparison of the TIC diagrams obtained under four different extraction methods. The differences in the TICs derived from the four extraction methods are clearly discernible. For the samples extracted via ultrasonic-assisted extraction with *n*-hexane and acetone, only a small number of compounds were detected, with 18 and 21 compounds detected respectively, as presented in Table 2. In contrast, when SPME is employed, most of the TIC peaks are well separated in the signal. By setting a matching degree of ≥ 80, potentially existing volatile compounds were screened, and a total of 59 compounds were identified.  Figure 1(c) depicts the compound component histogram under different extraction methods. Except for aliphatic compounds, the number of compounds measured by the SPME method is higher than that measured by other methods. Moreover, in the research on the medicinal components of Lauraceae plants, terpenoids and aldehyde compounds play more significant roles, and the SPME method can acquire more compound information with a smaller sample quantity. Overall, it is more advisable to select SPME as the optimal extraction method.

### 3.2. Screening of Different Fiber

The TIC profiles obtained from different fiber coatings are quite similar in terms of peak appearance. As shown in Figure 1(b), the 75-μm CAR/PDMS fiber was able to adsorb a greater number of volatile compounds.  Table 3 lists the number of compounds and total peak area of separated components obtained with different extraction fibers. Moreover, by comparing the number of compounds captured and the total peak area across the four fiber coatings, the 75-μm CAR/PDMS fiber detected 62 compounds, with a total peak area significantly higher than those of the other three fibers. Therefore, the 75-μm CAR/PDMS fiber was selected as the SPME fiber for sample extraction.

### 3.3. Optimization of the Single-Factor Test Conditions

Before the analysis, the adsorption conditions of SPME were also optimized. Single-factor experiments were carried out on three parameters: extraction time, extraction temperature, and equilibrium time.

#### 3.3.1. Selection of Different Extraction Temperatures

**Figure 2:**
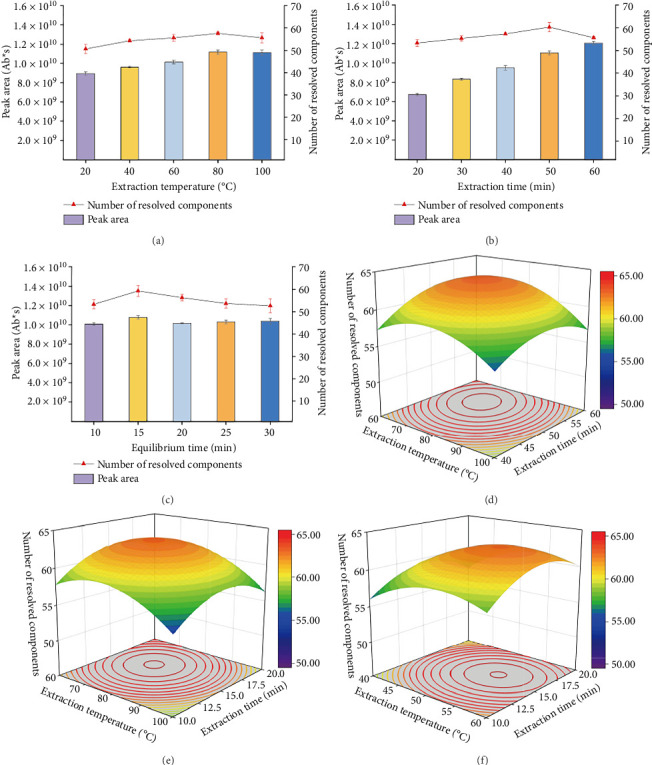
Screening of single-factor conditions and results of response surface data. (a) Scatter plot of the number of compounds and bar chart of the total peak area at different extraction temperatures. (b) Scatter plot of the number of compounds and bar chart of the total peak area at different extraction times. (c) Scatter plot of the number of compounds and bar chart of the total peak area at different equilibrium times. (d) Interaction response surface of extraction temperature and extraction time. (e) Interaction response surface of extraction temperature and equilibrium time. (f) Interaction response surface of extraction time and equilibrium time.

The extraction temperature has a significant impact on the extraction process. As shown in Figure 2(a), the total peak area of the compounds increases with the rise in extraction temperature. It is worth noting that although higher temperatures result in a larger total peak area, a decrease in the number of detectable compounds occurs after the temperature reaches 80°C. To further validate this observation, we conducted experiments at 120°C, detecting only 52 compounds. Moreover, temperatures exceeding 80°C generate significant amounts of water vapor during sample preparation, hindering the experimental process. Therefore, we conclude that 80°C is the likely optimal temperature condition.

#### 3.3.2. Selection of Different Extraction Times

Figure 2(b) shows the number of compounds and total peak area under different extraction times. The optimal extraction effect was observed at 50 min. A comparison of the number of detected compounds and total peak area shows that 58 compounds were detected at 50 min, with a total peak area of 1.13 × 10^10^, both representing the highest values among the five conditions tested. Therefore, 50 min was selected as the optimal extraction time.

#### 3.3.3. Selection of Different Equilibration Times

As shown in Figure 2(c), the optimal equilibration time was reached at 15 min. After 15 min, both the number of compounds and the total peak area gradually declined, likely due to prolonged heat exposure leading to compound degradation. Therefore, 10 min was selected as the optimal equilibration time.

### 3.4. Optimization of the Response Surface Test

A response surface experiment was designed using Design Expert 8.0.6, with a total of 17 experimental points, including 12 factorial experiments and 5 center points. The results are shown in Table 4.

A multiple regression analysis was conducted using Design Expert 8.0.6 on the experimental data, yielding a quadratic polynomial regression model for the volatile components of *Cinnamomi ramulus* as a function of extraction temperature (A), extraction time (B), and equilibration time (C). The resulting regression equation is *Y* = −80.2 + 1.26*A* + 2.625*B* + 3.43*C* − 0.00375*AB* − 0.0025*AC* + 0.005*BC* − 0.006937*A*^2^ − 0.02275*B*^2^ − 0.111*C*^2^. An analysis of variance (ANOVA) was performed on the regression model and its contributing factors, with the results summarized in Table 5.

As shown in Table 5, the *p* value of the model is less than 0.0001, indicating that the regression model is highly significant (*p* < 0.01). Meanwhile, the lack of fit term has a *p* value of 0.6137, which is not significant (*p* > 0.05), confirming the good fit of the regression equation. The model's coefficients of determination *R*^2^ = 0.9760 and *R*_Ad_^2^ = 0.9452 demonstrate a high degree of fit between the regression equation and the actual data. Therefore, this model can be used to analyze and predict the extraction of volatile components from Lamiaceae plants using the HS-SPME-GC-MS method. Additionally, the linear terms A and B, along with the quadratic terms *A*^2^, *B*^2^, *C*^2^ have highly significant effects on the extraction efficiency of volatile substances (*p* < 0.01), whereas term *C* shows a significant effect (*p* < 0.05). The ranking of the three factors in terms of their influence on the total peak area of volatile components is: extraction temperature (*A*) > extraction time (*B*) > equilibration time (*C*). The response surface results for the pairwise interactions between these factors are shown in Figures 2(d), 2(e), 2(f).

The prediction of the regression model gave the best condition for HS-SPME-GC-MS analysis of 0.5 g of samples equilibrated at 70.17°C for 14.7 min and extracted for 57.41 min.

### 3.5. Modeling Verification

**Figure 3:**
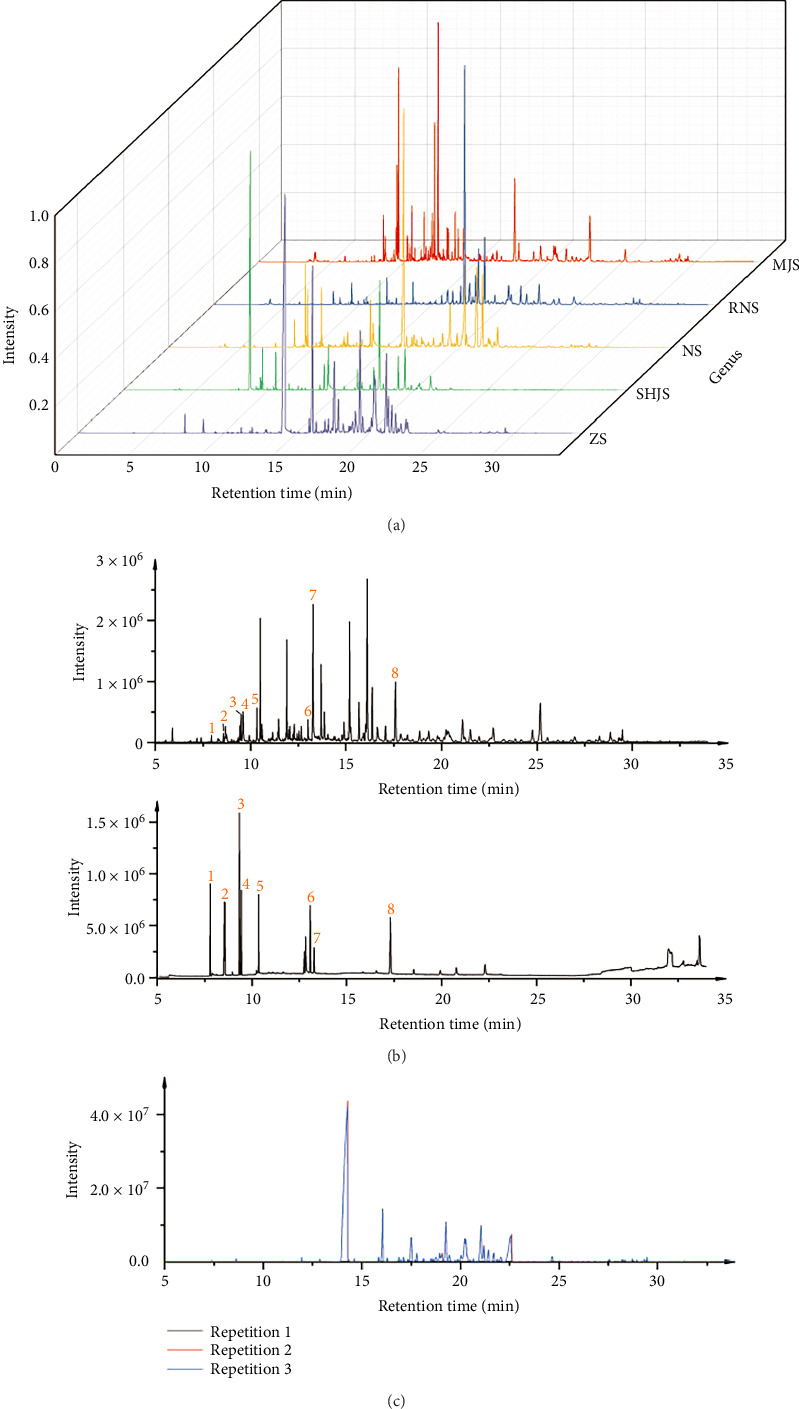
Peak emergence of samples and verification. (a) TIC diagrams of representative plants from five genera. (b) Component verification based on eight standard substances (1–8 are α-pinene, β-pinene, D-limonene, eucalyptol, linalool, D-carvone, citral, and trans-caryophyllene, respectively). (c) Repeatability verification.

As can be seen from Figure 3(a), this optimized condition is applicable to most Lauraceae plants. Judging from the representative plant samples of five genera currently tested, the peak shape is good and the distribution is uniform. As shown in Figures 3(b), 3(c), they are the qualitative result verification and repeatability verification of some standards, respectively. The qualitative results are accurate and the repeatability is good. Since both temperature and time need to be rounded, for the convenience of the experiment, the response surface test model was modified and verified according to the actual conditions. The extraction temperature was selected as 70°C, and after 15 min of equilibration, extraction was carried out for 57 min. Under these conditions, a total of 62 volatile substances could be detected finally, indicating that this mathematical model has good predictability and accuracy. Finally, this condition was selected as the optimal HS-SPME extraction condition.

### 3.6. Differential Analysis of Volatile Components in Samples From Five Different Genera

**Figure 4:**
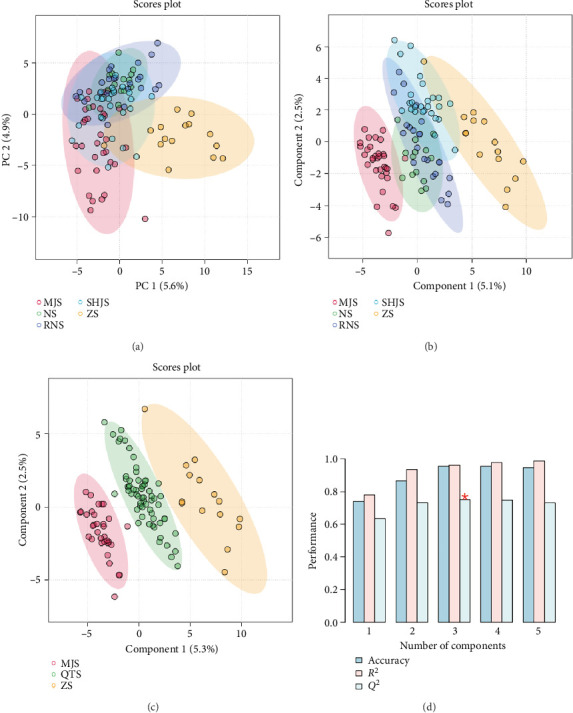
Chemometric analysis of samples from different genera and species in lauraceae. (a) PCA score plot, (b) PLS-DA score plot, (c) grouped PLS-DA, and (d) cross-validation.

The plant samples of five genera in the Lauraceae family are mainly composed of sesquiterpenoids and monoterpenoids. The peak areas of the samples from the five genera (with three replicates for each sample, and the average value was used for input) were selected for chemometric analysis. The results are shown in Figure 4(a). From the PCA, it can be found that due to the large differences in compounds, the samples of the *Litsea* genus and the *Cinnamomum* genus are evenly distributed on the positive and negative semi-axes, while the *Phoebe* genus, *Machilus* genus, and *Lindera* genus are clustered together, indicating that these three genera have a relatively high similarity compared with the *Litsea* genus and the *Cinnamomum* genus. The PLS-DA results in Figure 4(b) are also quite similar. Therefore, these three genera were grouped into one category, named “other category”, and PLS-DA analysis was carried out on the newly generated three groups of samples, as shown in Figure 4(c). The three groups can be well distinguished. And as shown in Figure 4(d), the values of *R*^2^, *Q*^2^, and the accuracy demonstrate that the model is in good condition and the results are stable and reliable. Next, chemical classification will be carried out pairwise among the three groups. The following are the specific analysis results.

#### 3.6.1. Analysis of Differences Between *Litsea* Genus and *Cinnamomum* Genus

**Figure 5:**
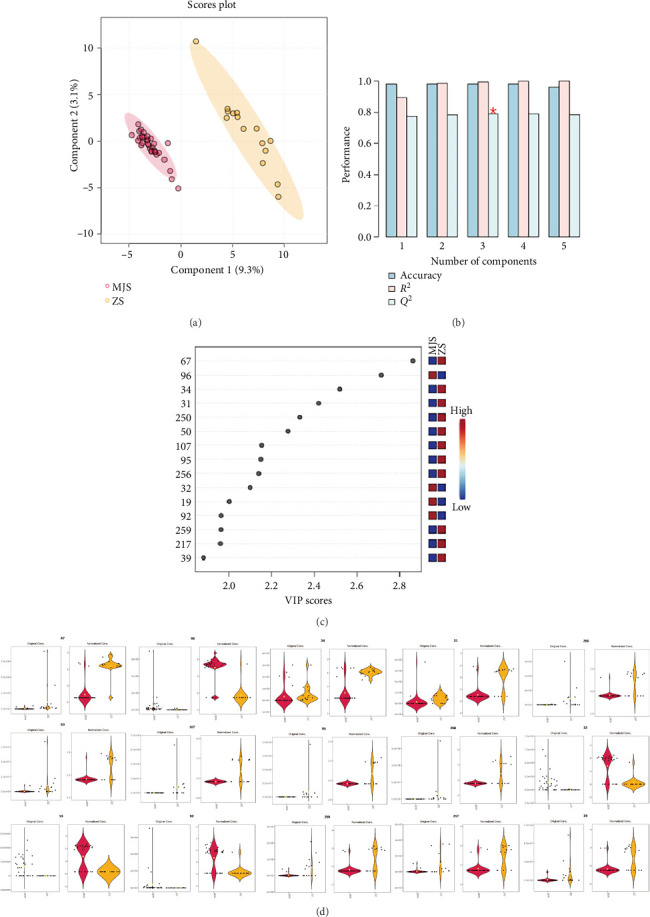
Chemometric analysis of *Litsea* and *Cinnamomum*. (a) PLS-DA score plot. (b) Cross-validation results. (c) VIP score plot. (d) Violin plots showing distribution of the top 15 differential metabolites.

Further comparative analysis was performed on the volatile components of the branch parts of 33 species of the *Litsea* genus and 16 species of the *Cinnamomum* genus, and the results are shown in Figure 5. The PLS-DA analysis diagram in Figure 5(a) clearly shows that there are significant differences between the two genera. This indicates that in the supervised two-dimensional analysis, there is still a large difference between the *Litsea* genus and the *Cinnamomum* genus, which is consistent with the previous PCA results, and according to the cross-validation results shown in Figure 5(b), no overfitting phenomenon was observed. The relatively concentrated aggregation of *Litsea* genus samples proves that there is little difference in the composition of the *Litsea* genus itself. Although the *Cinnamomum* genus can be distinguished from the *Litsea* genus, it can be found to be more dispersed in the vertical direction, so there are relatively large differences within the *Cinnamomum* genus.

According to the PLS-DA, there are 70 compounds with VIP > 1.  Figure 5(c) shows the top 15 compounds with VIP scores, which are candidate compounds for distinguishing between the *Litsea* genus and the *Cinnamomum* genus. The top 15 compounds were selected as the finally screened differential compounds, and the specific information is shown in Table 6.

The sample distribution of 15 differential metabolites was analyzed, and the results are shown in the violin diagram in Figure 5(d). Hexene, D-limonene, geraniol, and carvone are the volatile components unique to the *Litsea* genus compared with the *Cinnamomum* genus. This is consistent with the odor characteristics of the *Litsea* genus and the *Cinnamomum* genus. For example, the common Chinese herbal medicine *L. cubeba* of the *Litsea* genus has a strong lemon fragrance. Different from the lemon flavor of the *Litsea* genus, the *Cinnamomum* genus has a strong camphor odor. Compounds such as camphor, eucalyptol, p-cymene, benzyl benzoate, (Z)-p-2-menth-1-ol, safrole, cuminaldehyde, benzyl salicylate, ethyl palmitate, guaiol, and β-terpineol are the compounds that distinguish the *Cinnamomum* genus from other genera.

#### 3.6.2. Analysis of Differences Between the *Litsea* Genus and Other Genera

**Figure 6:**
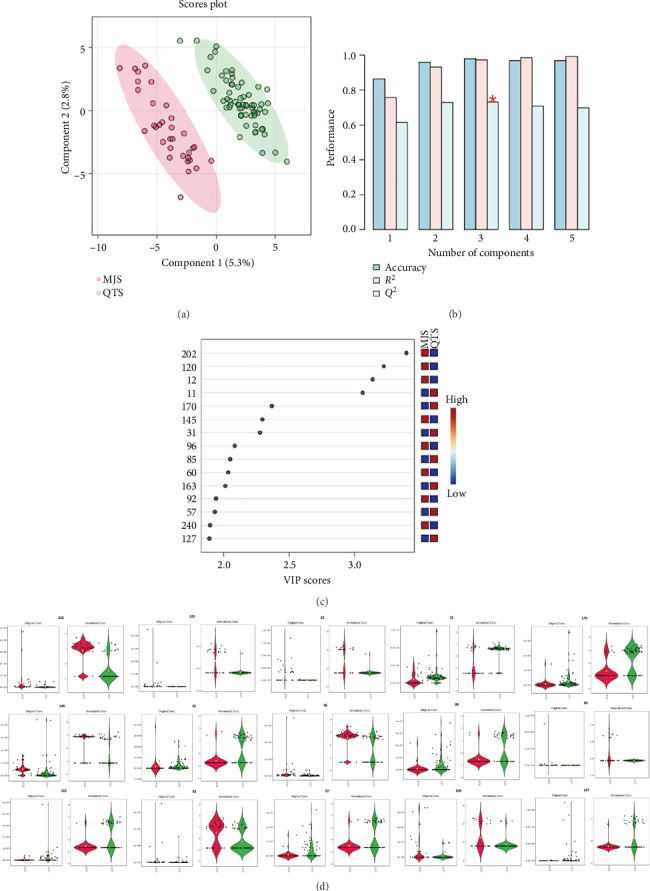
Chemometric analysis of *Litsea* and other genera. (a) PLS-DA score plot. (b) Cross-validation results. (c) VIP score plot. (d) Violin plots showing distribution of the top 15 differential metabolites.

Further analysis was conducted on 33 species of the *Litsea* genus and 63 species of other genera, and the results are shown in Figure 6(a). The PLS-DA analysis diagram in the above figure clearly shows that there are significant differences between the two genera. Moreover, Figure 6(b) demonstrates favorable cross-validation results.

According to the PLS-DA analysis in Figure 6(c), a total of 75 compounds had VIP > 1. The top 15 compounds with the highest VIP scores were selected as differential metabolites to distinguish between the two sample groups, with detailed information provided in Table 7.

As shown in Figure 6(d), it is the violin plot of the *Litsea* genus and other genera. Among them, nerolidol is widely distributed in the *Litsea* genus and can be used to distinguish the two types of samples. Nerolidol, carvyl acetate, carvone, verbenone, and α-bisabolol can be used to distinguish the other three genera. Isoledene, p-cymene, β-cubebene, campholenaldehyde, and calamenene are commonly found in the *Phoebe* genus, *Machilus* genus, and *Lindera* genus and can be distinguished from the *Litsea* genus.

#### 3.6.3. Chemical Difference Analysis Between the *Cinnamomum* Genus and Other Genera

Differential compounds are screened based on the VIP values and *p* values obtained from the PLS-DA model. According to the criteria of VIP > 1 and *p* < 0.05, 15 main differential compounds are finally obtained, most of which are aliphatic compounds, mainly including *n*-hexane and *n*-undecanal. The specific information of the compounds is shown in Table 8.

**Figure 7:**
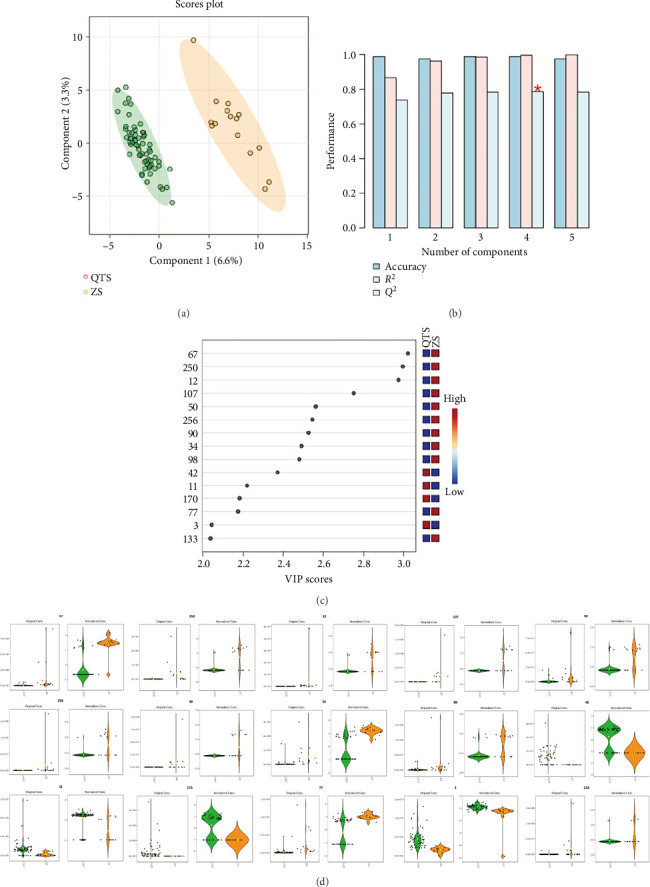
Chemometric analysis of *Cinnamomum* and other genera. (a) PLS-DA score plot. (b) Cross-validation results. (c) VIP score plot. (d) Violin plots showing distribution of the top 15 differential metabolites.

As shown in Figure 7, it is the comparison among the *Cinnamomum* genus and the *Lindera* genus, *Machilus* genus, and *Phoebe* genus. It can be seen that the difference between the *Cinnamomum* genus and the other genera in the figure is obvious. Most of the differential metabolites of the other genera are mainly aliphatic compounds, indicating that the other three genera have a better enrichment of aliphatic compounds compared with the *Cinnamomum* genus, while the *Cinnamomum* genus is rich in more oxygen-containing monoterpenoid compounds. However, since there are certain differences among the three genera themselves, a rather polarized difference is shown in the violin plot. If one wants to explore the differences among these three genera, further analysis is required.

#### 3.6.4. Analysis of Differences Among Other Genera

**Figure 8:**
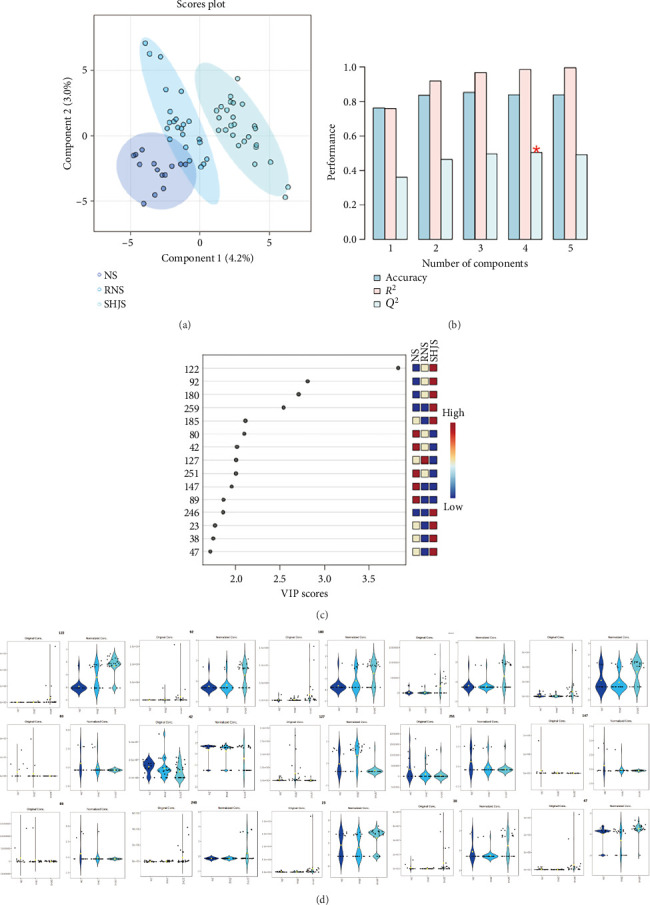
Chemometric analysis of the other three genera. (a) PLS-DA score plot. (b) Cross-validation results. (c) VIP score plot. (d) Violin plots showing distribution of the top 15 differential metabolites.

As shown in Figure 8(a), a supervised analysis was performed on the three genera, and it was found that the *Machilus* genus and the *Phoebe* genus are closer to each other, while the *Lindera* genus was distinguished. As shown in Figures 8(b), 8(c), according to the criteria of VIP > 1 and *p* < 0.05, 80 main differential compounds were finally obtained, and the top 15 compounds were selected by VIP score ranking, mainly including oxygen-containing monoterpenoids and sesquiterpenoids. The specific compound information is shown in Table 9. According to the violin plot to explore the distribution of differential components among the three genera, it can be found that there is a large gap between the *Lindera* genus and the *Phoebe* genus and the *Machilus* genus, especially geranyl acetate, geraniol, β-elemene, β-selinene, and linalyl geranyl acetate. It indicates that the *Lindera* genus contains more oxygen-containing monoterpenoid compounds compared with the *Phoebe* genus and the *Machilus* genus. Myrtanal, calamenene, guaiol, isocaryophyllene, and 2-isopropyl-5-methylanisole have a relatively high content distribution in the *Phoebe* genus and the *Machilus* genus. Calamenene and guaiol are also the differential metabolites between them and the *Cinnamomum* genus and the *Litsea* genus, indicating that such compounds may serve as the differential metabolites that distinguish the *Phoebe* genus and the *Machilus* genus from other genera.

#### 3.6.5. Comparison of Differential Metabolites

**Figure 9:**
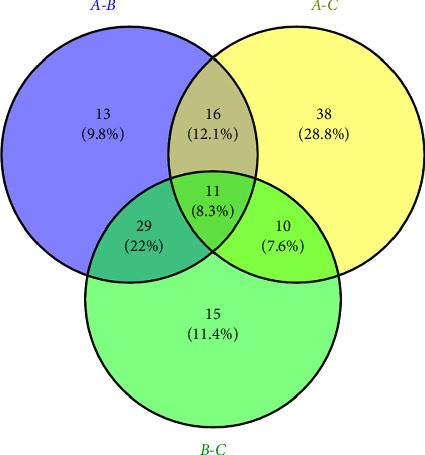
Venn diagram of differential metabolites of each genus.

The differential metabolites of each group were aggregated, and the landmark differential metabolites among each genus were searched through Figure 9, Venn diagrams. Detailed compounds can be found in Table 10. There are a total of eight common compounds, including carvone and ethyl palmitate. There are 19 unique compounds in the *Litsea* genus, such as D-limonene, geraniol, and nerolidol. While the *Cinnamomum* genus has 27 landmark differential metabolites, such as camphor, eucalyptol, and safrole, there are 10 differential metabolites in other genera, such as styrene, *n*-heptanal, and isoledene. It can be seen that there are huge differences in volatile components among the genera of Lauraceae plants. Compared to trait-based differential analysis [26], this method provides an in-depth analysis of five genera within the Lauraceae family from a chemical composition perspective. Currently known detection methods for the classification of Lauraceae plants often rely on UV and nuclear magnetic resonance (NMR) techniques [27]. In comparison, the HS-SPME-GC-MS detection method offers greater convenience and cost-effectiveness. This method enables the rapid detection of compound compositions in various samples and utilizes differential compounds to effectively distinguish among different genera. Furthermore, the unique chemical composition profiles can provide a significant material basis for the further development and utilization of plants within each genus.

## 4. Conclusion

This study is based on the established HS-SPME, combined with gas chromatography-mass spectrometry. Eventually, 75-μm CAR/PDMS was determined as the optimal adsorption fiber coating. Taking 0.500 g of the sample, after equilibration for 15 min, extraction at 70°C for 57 min can yield better results. Representative plant samples of five genera were verified and subjected to chemometric analysis, and differential metabolites that can be used to distinguish each genus were found, proving that the genera of Lauraceae can be distinguished by compounds and confirming the feasibility of chemical classification. This optimized content provides a relatively practical experimental condition for the analysis of volatile components of Lauraceae plants and also provides certain help for further chemical classification of volatile components of Lauraceae plants and the development of nonmedicinal plants.

## Figures and Tables

**Table 1:** The factors and levels of response surface test.

Level	Factor		
	*A*: Extraction temperature (°C)	*B*: Extraction time (min)	*C*: Equilibrium time (min)
−1	50	30	10
0	60	40	15
1	70	50	20

**Table 2:** The number and total peak area of resolved components under different extraction conditions.

Extraction method	Number of resolved components	Total peak area
*n*-Hexane	18	1.97 × 10^8^
Acetone	21	5.84 × 10^8^
HS-SPME	59	1.14 × 10^10^
Volatile oil	42	1.36 × 10^10^

**Table 3:** The number and total peak area of resolved components under different extraction fibers.

Extraction fiber	Number of resolved components	Total peak area
50/30-μm DVB/CAR/PDMS	54	8.67 × 10^9^
65-μm PDMS/DVB	55	7.45 × 10^9^
100-μm PDMS	54	5.69 × 10^9^
75-μm CAR/PDMS	62	1.04 × 10^10^

**Table 4:** Response surface test design and results.

Std	Run	*A* (°C)	*B* (min)	*C* (min)	Number of resolved components
1	17	−1 (60)	−1 (40)	0 (15)	57
2	14	1 (100)	−1	0	56
3	8	−1	1 (60)	0	61
4	16	1	1	0	57
5	2	−1	0 (50)	−1 (10)	58
6	10	1	0	−1	55
7	4	−1	0	1	60
8	5	1	0	1	56
9	12	0 (80)	−1	−1	56
10	6	0	1	−1	58
11	3	0	−1	1 (20)	57
12	9	0	1	1	60
13	11	0	0	0	64
14	13	0	0	0	62
15	7	0	0	0	63
16	1	0	0	0	62
17	15	0	0	0	63

**Table 5:** Variance analysis of regression equation of response surface test.

Source	Sum of squares	df	Mean square	F-value	*p* value	Significance
Model	61.55	9	14.94	31.69	< 0.0001	^∗∗^
*A*	4.91	1	18	38.18	0.0005	^∗∗^
*B*	1.92	1	12.5	26.52	0.0013	^∗∗^
*C*	1.47	1	4.5	9.55	0.0176	^∗^
*AB*	0.038	1	2.25	4.77	0.0652	
*AC*	1.17	1	0.25	0.5303	0.4901	
*BC*	0.0006	1	0.25	0.5303	0.4901	
*A* ^2^	24.97	1	32.42	68.78	< 0.0001	^∗∗^
*B* ^2^	5.54	1	21.79	46.23	0.0003	^∗∗^
*C* ^2^	16.59	1	32.42	68.78	< 0.0001	^∗∗^
Residual	2.73	7	0.4714			
Lack of fit	0.9056	3	0.1667	0.2381	0.866	Not significant
Pure error	1.82	4	0.7			
Cor total	64.27	16				
*R* ^2^ = 0.9760 *R*_Adj_^2^ = 0.9452 *R*_Pre_^2^ = 0.9102 C.V. = 1.16%						

^∗^A significant difference (*p* < 0.05).

^∗∗^An extremely significant difference (*p* < 0.01).

**Table 6:** 15 Differential metabolites in the genera *Litsea* and *Cinnamomum*.

No.	Chemical compound	Formula	Classification	VIP value
1	(+)-2-Bornanone	C_10_H_16_O	Oxygenated monoterpenoids	2.861
2	D-Carvone	C_10_H_16_O	Oxygenated monoterpenoids	2.7131
3	Eucalyptol	C_10_H_18_O	Oxygenated monoterpenoids	2.5178
4	p-Cymene	C_10_H_14_	Aromatics	2.4202
5	Benzyl benzoate	C_14_H_12_O_2_	Aromatics	2.3318
6	(Z)-para-2-Menthen-1-ol	C_10_H_18_O	Oxygenated monoterpenoids	2.2769
7	Safrole	C_10_H_10_O_2_	Aromatics	2.1535
8	4-Isopropylbenzaldehyde	C_10_H_12_O	Aromatics	2.1494
9	Benzylsalicylate	C_14_H_12_O_3_	Aromatics	2.1391
10	D-Limonene	C_10_H_16_	Monoterpenoids	2.0988
11	Hexanoic acid	C_6_H_12_O_2_	Aliphatics	2.0011
12	Geraniol	C_10_H_18_O	Oxygenated monoterpenoids	1.9629
13	Ethylpalmitate	C_15_H_30_O_2_	Aliphatics	1.9625
14	Guaiol	C_15_H_26_O	Oxygenated sesquiterpenoids	1.9595
15	β-Terpineol	C_10_H_18_O	Oxygenated monoterpenoids	1.8817

**Table 7:** 15 differential metabolites between the genus *Litsea* and other genera.

No.	Chemical compound	Formula	Classification	VIP value
1	Nerolidol	C_15_H_26_O	Oxygenated sesquiterpenoids	3.3973
2	Carveol acetate	C_12_H_18_O_2_	Oxygenated monoterpenoids	3.2243
3	Styrene	C_8_H_8_	Aromatics	3.1373
4	Heptanal	C_7_H_14_O	Aliphatics	3.062
5	Isoledene	C_15_H_24_	Sesquiterpenoids	2.3654
6	(E)-β-Farnesene	C_15_H_24_	Sesquiterpenoids	2.2928
7	p-Cymene	C_10_H_14_	Aromatics	2.275
8	D-Carvone	C_10_H_16_O	Oxygenated monoterpenoids	2.0832
9	Verbenone	C_10_H_14_O	Oxygenated monoterpenoids	2.0482
10	(R)-(+)-Citronellol	C_10_H_18_O	Oxygenated monoterpenoids	2.0336
11	β-Cubebene	C_15_H_24_	Sesquiterpenoids	2.0119
12	Geraniol	C_10_H_18_O	Oxygenated monoterpenoids	1.9406
13	(R)-α-Campholenaldehyde	C_10_H_16_O	Oxygenated monoterpenoids	1.9319
14	α-Bisabolol	C_15_H_24_	Oxygenated sesquiterpenoids	1.8935
15	(+)-Calarene	C_15_H_24_	Sesquiterpenoids	1.8882

**Table 8:** 15 Differential metabolites between the genus *Cinnamomum* and other genera.

No.	Chemical compound	Formula	Classification	VIP value
1	(+)-2-Bornanone	C_10_H_16_O	Oxygenated monoterpenoids	3.0211
2	Benzyl benzoate	C_14_H_12_O_2_	Aromatics	2.9954
3	Styrene	C_8_H_8_	Aromatics	2.9737
4	Safrole	C_10_H_10_O_2_	Aromatics	2.7502
5	(Z)-para-2-Menthen-1-ol	C_10_H_18_O	Oxygenated monoterpenoids	2.5621
6	Benzyl salicylate	C_14_H_12_O_3_	Aromatics	2.5455
7	(Z)-Citral	C_10_H_16_O	Oxygenated monoterpenoids	2.5255
8	Eucalyptol	C_10_H_18_O	Oxygenated monoterpenoids	2.4909
9	(E)-Citral	C_10_H_16_O	Oxygenated monoterpenoids	2.4802
10	Undecane	C_11_H_24_	Aliphatics	2.3714
11	Heptanal	C_7_H_14_O	Aliphatics	2.2188
12	Isoledene	C_15_H_24_	Sesquiterpenoids	2.1818
13	α-Terpineol	C_10_H_18_O	Oxygenated monoterpenoids	2.1744
14	Hexanal	C_6_H_12_O	Aliphatics	2.0435
15	Methyleugenol	C_11_H_14_O_2_	Aromatics	2.0374

**Table 9:** 15 differential metabolites among the remaining three genera (Phoebe, Machilus, and Lindera).

No.	Chemical compound	Formula	Classification	VIP value
1	Geranyl acetate	C_12_H_20_O_2_	Oxygenated monoterpenoids	3.8288
2	Geraniol	C_10_H_18_O	Oxygenated monoterpenoids	2.8112
3	β-Selinene	C_15_H_24_	Sesquiterpenoids	2.7091
4	Ethyl palmitate	C_15_H_30_O_2_	Aliphatics	2.5423
5	α-Selinene	C_15_H_24_	Sesquiterpenoids	2.112
6	Myrtenal	C_10_H_14_O	Oxygenated monoterpenoids	2.0991
7	Undecane	C_11_H_24_	Aliphatics	2.0164
8	(+)-Calarene	C_15_H_24_	Sesquiterpenoids	2.0065
9	Guaiazulene	C_15_H_18_	Aromatics	2.0048
10	Isocaryophyllene	C_15_H_24_	Sesquiterpenoids	1.956
11	2-Isopropyl-5-methylanisole	C_11_H_16_O	Aromatics	1.8641
12	Eicosane	C_20_H_42_	Aliphatics	1.8594
13	β-Myrcene	C_10_H_16_	Monoterpenoids	1.7722
14	cis-Linaloloxide	C_10_H_18_O_2_	Oxygenated monoterpenoids	1.7528
15	Linalool	C_10_H_18_O	Oxygenated monoterpenoids	1.7187

**Table 10:** The specific and common metabolite compounds of different genus detected by HS-SPME-GC-MS in Lauraceae.

No.	*Litsea*(16)	*Cinnamomum*(29)	Others(10)	Common (11)
1	p-Cymene	Bornanone	Styrene	D-Carvone
2	Geraniol	Eucalyptol	Heptanal	D-Limonene
3	Nerolidol	Benzyl benzoate	Isoledene	Ethyl palmitate
4	α-Calacorene	(Z)-para-2-Menthen-1-ol	α-Himachalene	Guaiol
5	Carveol acetate	Safrole	α-Muurolene	α-Cubebene
6	Spathulenol	4-Isopropylbenzaldehyde	Linalool	Alloocimene
7	Humulene epoxide II	Benzyl salicylate	Carveol	Calarene
8	β-Elemene	Hexanoic acid	Octanal	Undecane
9	γ-Muurolene	β-Terpineol	β-Patchoulene	α-Terpineol
10	Hexadecane	Benzaldehyde	2-Carene	(Z)-Citral
11	Campholenaldehyde	Methyleugenol		(E)-Citral
12	Muurola-3,5-diene	Undecanoicacid		
13	α-Copaene	Hexanal		
14	α-Cedrene	α-Thujene		
15	Myrtenal	α-Cadinol		
16	Geranyl acetate	Tetradecanal		
17		Dodecene		
18		(+)-Camphene		
19		*para*-Menthatriene		
20		Nerol		
21		Eugenol		
22		1,5,5-Trimethyl-6-methylene-cyclohexene		
23		4-Carene		
24		1-Tetradecanol		
25		β-Neoclovene		
26		Myrcenol		
27		(E)-para-2-Menthen-1-ol		
28		β-Ionone		
29		Selina-3,7(11)-diene		

## Data Availability

Data are available upon request due to privacy/ethical restrictions.

## References

[1] Farias K. S., Alves F. M., Santos-Zanuncio V. S., de Sousa Jr P. T., Silva D. B., Carollo C. A. (2023). Global Distribution of the Chemical Constituents and Antibacterial Activity of Essential Oils in Lauraceae Family: A Review. *South African Journal of Botany*.

[2] Chau D. T. M., Chung N. T., Huong L. T. (2020). Chemical Compositions, Mosquito Larvicidal and Antimicrobial Activities of Leaf Essential Oils of Eleven Species of Lauraceae From Vietnam. *Plants*.

[3] Cardoso E. K. S., Kubota K., Luz D. A. (2022). *Aniba canelilla* (Kunth) *Mez* (Lauraceae) Essential Oil: Effects on Oxidative Stress and Vascular Permeability. *Antioxidants*.

[4] Gonçalves Vasconcelos de Alcântara B., Neto A. K., Garcia D. A. (2023). Anti-Inflammatory Activity of Lauraceae Plant Species and Prediction Models Based on their Metabolomics Profiling Data. *Chemistry and Biodiversity*.

[5] D’Aquila P., Sena G., Crudo M., Passarino G., Bellizzi D. (2023). Effect of Essential Oils of Apiaceae, Lamiaceae, Lauraceae, Myrtaceae, and Rutaceae Family Plants on Growth, Biofilm Formation, and Quorum Sensing in *Chromobacterium violaceum*, *Pseudomonas aeruginosa*, and *Enterococcus faecalis*. *Microorganisms*.

[6] Bora H., Kamle M., Hassan H. (2022). Exploration of Potent Antiviral Phytomedicines From Lauraceae Family Plants Against SARS-CoV-2 Main Protease. *Viruses*.

[7] Rabaan A. A., Halwani M. A., Aljeldah M. (2023). Exploration of Potent Antiviral Phytomedicines From Lauraceae Family Plants Against SARS-CoV-2 RNA-Dependent RNA Polymerase. *Journal of Biomolecular Structure and Dynamics*.

[8] Tan C., Ferguson D. K., Tang Z., Yang Y. (2023). Distribution and Conservation of the Lauraceae in China. *Global Ecology and Conservation*.

[9] Tian Y., Zhou J., Zhang Y. (2021). Research Progress in Plant Molecular Systematics of Lauraceae. *Biology*.

[10] Liu X., Yang J., Fu J. (2018). Phytochemical and Chemotaxonomic Studies on the Twigs of *Cinnamomum* Cassia (Lauraceae). *Biochemical Systematics and Ecology*.

[11] Silva E Silva Figueiredo C. S., de Oliveira P. V., Dos Reis Ferreira L. (2023). Cinnamaldehyde for the Treatment of Microbial Infections: Evidence Obtained From Experimental Models. *Current Medicinal Chemistry*.

[12] Huong L. T., Chau D. T. M., An N. T. G., Dai D. N., Giwa-Ajeniya A. O., Ogunwande I. A. (2024). Essential Oils of Lauraceae: Antimicrobial Activity and Constituents of Essential Oil From Two *Machilus* Species From Vietnam. *Journal of Essential Oil Bearing Plants*.

[13] Azadi A., Rafieian F., Sami M., Rezaei A. (2023). Fabrication, Characterization and Antimicrobial Activity of Chitosan/Tragacanth Gum/Polyvinyl Alcohol Composite Films Incorporated With Cinnamon Essential Oil Nanoemulsion. *International Journal of Biological Macromolecules*.

[14] Caserta S., Genovese C., Cicero N., Gangemi S., Allegra A. (2023). The Anti-Cancer Effect of Cinnamon Aqueous Extract: A Focus on Hematological Malignancies. *Life (Basel)*.

[15] Han X., Zhou W., Zhang J. (2023). *Lindera* lactone Mitigates Diabetic Cardiomyopathy in Mice via Suppressing the MAPK/ATF6 Pathway. *International Immunopharmacology*.

[16] Trung T. Q., Hiep H. P., Van Khang P. (2023). Chemical Compositions of *Litsea* umbellata and Inhibition Activities. *Open Chemistry*.

[17] Anzano A., de Falco B., Grauso L., Motti R., Lanzotti V. (2022). Laurel, *Laurus nobilis L.*: A Review of Its Botany, Traditional Uses, Phytochemistry and Pharmacology. *Phytochemistry Reviews*.

[18] Teng H., Lee W. Y. (2014). Antibacterial and Antioxidant Activities and Chemical Compositions of Volatile Oils Extracted From Schisandra chinensis Baill. Seeds Using Simultaneous Distillation Extraction Method, and Comparison With Soxhlet and Microwave-Assisted Extraction. *Bioscience, Biotechnology, and Biochemistry*.

[19] Ruiz-Jimenez J., Raskala S., Tanskanen V. (2023). Evaluation of Vocs From Fungal Strains, Building Insulation Materials and Indoor Air by Solid Phase Microextraction Arrow, Thermal Desorption–Gas Chromatography-Mass Spectrometry and Machine Learning Approaches. *Environmental Research*.

[20] Ma X. L., Wang X. C., Zhang J. N. (2023). A Study of Flavor Variations During the Flaxseed Roasting Procedure by Developed Real-Time SPME GC-MS Coupled With Chemometrics. *Food Chemistry*.

[21] Xie J., Wang L., Deng Y. (2023). Characterization of the Key Odorants in Floral Aroma Green Tea Based on GC-E-Nose, GC-IMS, GC-MS and Aroma Recombination and Investigation of the Dynamic Changes and Aroma Formation During Processing. *Food Chemistry*.

[22] Jiang K., Xu K., Wang J., Meng F., Wang B. (2023). Based on HS-SPME-GC-MS Combined With GC-O-MS to Analyze the Changes of Aroma Compounds in the Aging Process of Citri Reticulatae Pericarpium. *Food Bioscience*.

[23] Qi S., Zha L., Peng Y. (2022). Quality and Metabolomics Analysis of *Houttuynia cordata* Based on HS-SPME/GC-MS. *Molecules*.

[24] Shao Y., Liu X., Zhang Z., Wang P., Li K., Li C. (2023). Comparison and Discrimination of the Terpenoids in 48 Species of Huajiao According to Variety and Geographical Origin by E-Nose Coupled With HS-SPME-GC-MS. *Food Research International*.

[25] Wang X., Zhong L., Zou X. (2023). GC-MS and UHPLC-QTOFMS-Assisted Identification of the Differential Metabolites and Metabolic Pathways in Key Tissues of Pogostemon Cablin. *Frontiers in Plant Science*.

[26] Yang Z., Liu B., Yang Y., Ferguson D. K. (2022). Phylogeny and Taxonomy of *Cinnamomum* (Lauraceae). *Ecology and Evolution*.

[27] Lian M.-Y., Zhang Y.-J., Dong S.-H., Huang X.-X., Bai M., Song S.-J. (2022). Terpenoids From *Litsea lancilimba* Merr. and Their Chemotaxonomic Significant. *Biochemical Systematics and Ecology*.

